# Effects of selfsuperparasitism and host age on fitness-correlated traits in the solitary endoparasitoid wasp *Meteorus pulchricornis*

**DOI:** 10.1093/jis/14.1.103

**Published:** 2014-08-01

**Authors:** Bo Zhang, Baoping Li, Ling Meng

**Affiliations:** College of Plant Protection, Nanjing Agricultural University, Agricultural Pests Management Laboratory of Ministry of Education, Nanjing 210095, PR China

**Keywords:** development, fitness, life history, koinobiont, superparasitism, parasitoids

## Abstract

The domed fitness functions are suggested to describe developmental patterns of progeny parasitoids in relation to host age at oviposition in solitary koinobint parasitoids that are engaged in single parasitism, but few studies have investigated the applicability of the functions as related to superparasitism. The present study was designed to compare fitness functions between single parasitism and superparasitism by examining developmental patterns of *Meteorus pulchricornis* (Wesmael) (Hymneoptera: Braconidae) progeny in relation to the beet armyworm, *Spodoptera exigua* Hübner (Lepidoptera: Noctuidae), either singly parasitized or self-superparasitized as second–fifth instar larvae. Selfsuperparasitism caused deleterious effects on the fitness-related traits of parasitoid progeny, as demonstrated by a prolonged egg-to-adult emergence time, a smaller body size, and shorter longevity of the emerging adults, and decreased survival to adult emergence. While the domed fitness function was detected for development time, survival, adult body size, and longevity in relation to host larvae that were singly parasitized, the function was observed only for progeny survival in relation to host larvae that were self-superparasitized. This study suggests that developmental fitness functions with selfsuperparasitism can deviate from those with single parasitism in solitary koinobiont parasitoids.

## Introduction

Among the most important trade-offs faced by organisms is whether to grow larger at the cost of extended development time or to develop more rapidly at the cost of reduced size ( Abrams and Rowe 1996 ). To understand factors influencing offspring development and host selection strategies in parasitoids, the vast majority of studies have focused on the effects of host age, stage, or mass at parasitism on developmental traits in their parasitoids (reviewed by Mackauer and Sequeira 1993 ; Godfray 1994 ). The studies observe that the fitness functions differ markedly between (and within) koinobiont and idiobiont parasitoids in response to ecophysiological characteristics of the host species, including host size, growth potential at parasitism, immunity, and feeding ecology ( Harvey et al. 2004 ; Pennacchio and Strand 2006 ). While a clearcut host size– parasitoid fitness relationship is recognized for idiobionts, it is not always the case for koinobionts. The relationship between host quality and parasitoid fitness is much more complicated than is the case with idiobionts because it is determined by a range of potentially interacting ecophysiological selection pressures that are less prevalent in the latter group of parasitoids. For koinobionts attacking hosts of limited growth potential relative to maximum parasitoid size, the pattern is similar to that observed in idiobionts ( Harvey et al. 1994 ). In contrast, fitness functions for solitary parasitoids attacking hosts with the potential to grow considerably larger than the adult parasitoid are distinctly dome-shaped (or may even decrease with host size in extreme cases) because progressively larger hosts possess more potent immunological defenses, or else they may be less nutritionally compatible for parasitoid development (and survival) than younger hosts ( Strand 2000 ; Harvey et al. 2004 ). In the dome-shaped developmental model, fitness for parasitoids is not a linear or increasing function of host size or age at parasitism, but initially increases with host size and then rapidly declines as resources increase beyond the capacity of the parasitoid larva to consume them. The model gains strong support in numerous studies of solitary parasitoids involved in single parasitism ( Smilowitz and Iwantsch 1975 ; Harvey et al. 1994 , 1999, 2004; Pandey and Singh 1999 ; Chau and Mackauer 2001 ; Elzinga et al. 2003 ; Harvey et al. 2004 ; Liu and Li 2006 , 2008; Chen et al. 2011 ), but few studies have yet investigated the applicability of this model to the parasitoids engaged in superparasitism.

As hosts represent a limited discrete resource, superparasitism generally results in reduction in survivorship of larvae and/or size, longevity, and fecundity of emerging adults ( Visser et al. 1992 ; Harvey et al. 1993 ). However, superparasitism commonly is found not only in the laboratory ( van Alphen and Marcel 1990 ; Bai and Mackauer 1992 ; Visser et al. 1992 ; Feuster et al. 1993; Yamada and Miyamoto 1998 ; Chau and Mackauer 1999 ), but in the field as well ( Janssen 1989 ; Santolamazza-Carbone and Rivera 2003 ; Fleury et al. 2004 ; Jarmillo et al. 2006; Zhang et al. 2012 ). Superparasitism is now generally interpreted as an adaptive strategy in numerous theoretic models and empirical studies ( van Alphen and Visser 1990 ; Visser et al. 1992 ; Mackauer and Chau 2001 ; Yamada and Kuzuma 2003 ; Reynolds and Hardy 2004 ) or as a result of virus manipulation ( Varaldi et al. 2003 ; Gandon et al. 2006). It is therefore necessary to investigate developmental fitness functions in relation to host age in solitary koinobionts that are engaged in superparasitism so as to fully understand developmental strategies of the parasitoid wasps.

This study was designed to investigate the re lationship between fitness-correlated traits of *Meteorus pulchricornis* (Wesmael) (Hymneoptera: Braconidae) progeny in either singly parasitized or self-superparasitized *Spodoptera exigua* Hübner (Lepidoptera: Noctuidae) hosts as second-fifth instar larvae, with the goal of testing whether the domed fitness functions apply to the parasitoid engaged in single as well as selfsuperparasitism. *M. pulchricornis* has a wide host range of exposed-living lepidopteran larvae ( Huddleston 1980 ; Maeto 1989 , 1990) and is a potential biological control agent against some lepidopteran pests ( Fuester et al. 1993 ; Liu and Li 2006 ; Chau and Maeto 2008 ; Wu et al. 2008 ). The host suitability for development of the parasitoid progeny varies with host instars at the time of oviposition ( Askari et al. 1977 ; Liu and Li 2006 , 2008). Although foraging parasitoids can avoid selfsuperparasitism on the basis of host movement ( Chau and Maeto 2009 ; Zhang et al. 2012 ), superparasitism rate still can be as high as 31% in the laboratory ( Fuester et al. 1993 ) and 23% in the field ( Zhang et al. 2012 ).

## Materials and Methods

### Culture of the parasitoid and host

*Meteorus pulchricornis* was obtained from *S. exigua* larvae collected in soybean fields and maintained using *S. exigua* as host in the insectary (26 ± 2ºC, 60%-80% RH, 14L:10D). The parasitoid is thelytokous. The host larvae were reared on an artificial diet ( Liu and Li 2006 ).

### Experiment

*Spodoptera exigua* larvae undergo five instars (designated as L1-L5) before pupation. As the first instar larva was not susceptible to selfsuperparasitism in the exploratory trial (n = 30), it was not used in the experiment. The exploratory trial (n = 50) indicated that females that realized one egg deposition performed a characteristic stinging behavior (flapping wings when withdrawing the ovipositor), which was used to obtain hosts that were either singly parasitized (1 egg) or superparasitized (2 eggs). Host larvae were individually exposed to a parasitoid in a glass vial and continuously observed until they were attacked once (single parasitism) or twice (selfsuperparasitism). The time interval between the first and second stinging was kept within 30 minutes; within this time, symmetrical competition between offspring larvae can be created in this parasitoid ( Chau and Maeto 2008 ). The host larvae treated above were individually reared on the artificial diet in vials and monitored daily for egression of mature larvae, pupation, and adult emergence of progeny parasitoids. The emerged adults were kept in groups without feeding and hosts until dead, by which longevity was measured. The development time of progeny parasitoids was measured from oviposition to adult emergence (egg-adult emergence time), and the survival from this period was recorded. The right hind tibia length of the adults (a standard measure of body size) was measured under a stereomicroscope. Data were obtained from between 50 and 70 hosts for each parasitism mode/instar combination.

### Statistical analysis

Two-way ANOVA was used to compare single parasitism and superparasitism in developmental parameters of offspring parasitoids. Orthogonal polynomial regression was used to analyze the trend in development time, body size, and longevity of offspring according to host stages with either single parasitism or selfsuperparasitism, with the goal of test on the quadratic term as the manifestation of the domed fitness functions. This regression method can overcome computational difficulties due to collinearity ( Armitage et al. 2002 ). The Cochran-Armitage test of trend was used to approximately test for departures from a non-monotonic, dome-shaped trend in survival proportions across host stages at oviposition for single parasitism or selfsuperparasitism. Analyses were carried out using R ( R Development Core Team 2009 ).

## Results

**Figure 1.**
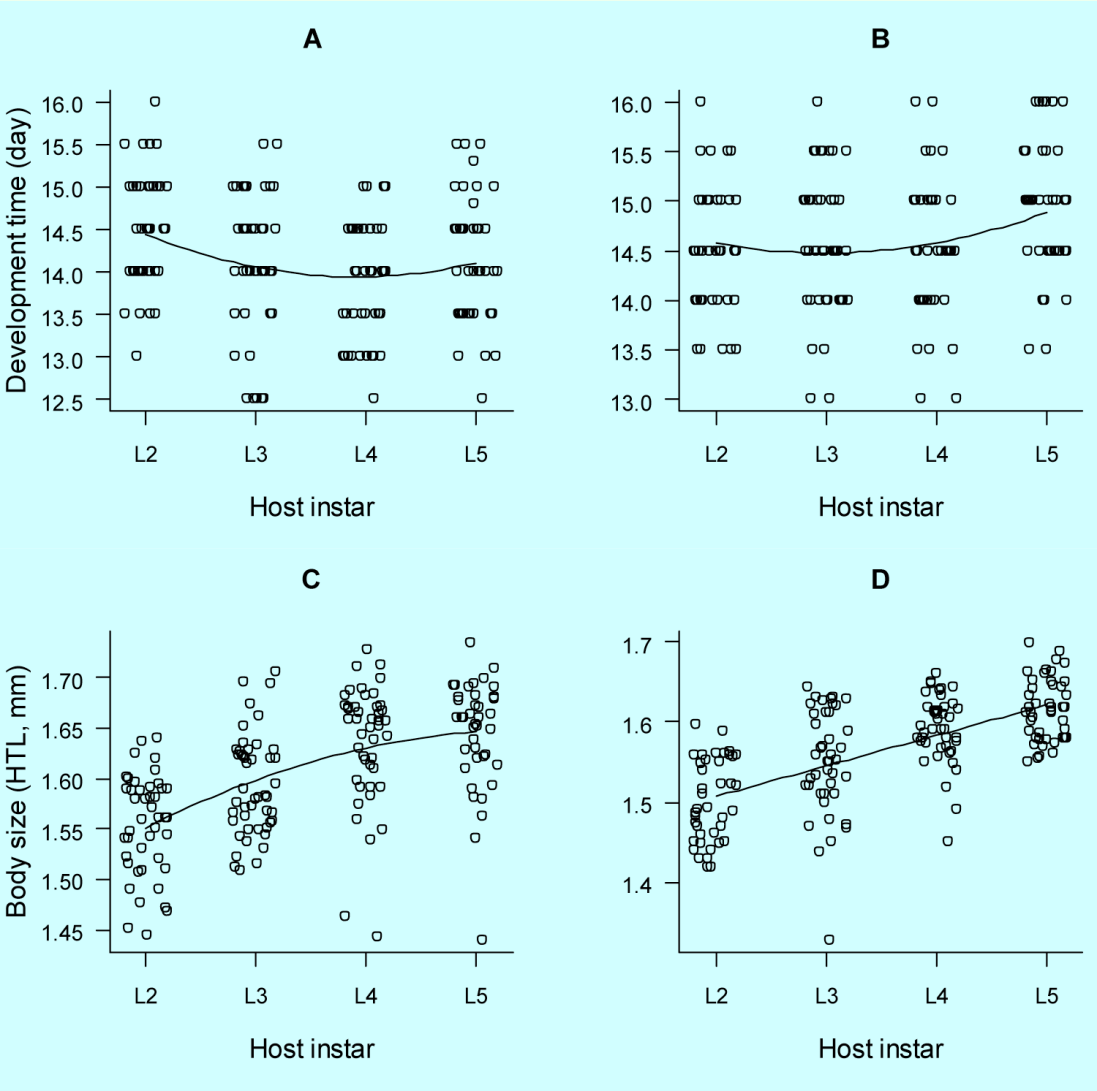
*Meteorus pulchricornis* development time (egg-adult emergence) and body size (hind tibia length, HTL) in relation to host instar singly parasitized (A, C) and self-superparasitized (B, D) as second-fifth instar larvae. High quality figures are available online.

A two-way ANOVA on development time revealed a significant effect of parasitism mode, where offspring parasitoids extended the egg-to-adult emergence time in self-superparasitized host larvae as opposed to counterparts in singly parasitized hosts ( Figure 1 ). Orthogonal polynomial regression analysis revealed best fits of a quadratic curvilinear function to the relationship between development time and host stage at both single parasitism and selfsuperparasitism ( Figure 1A , B; Table 1 ).

**Table 1.**
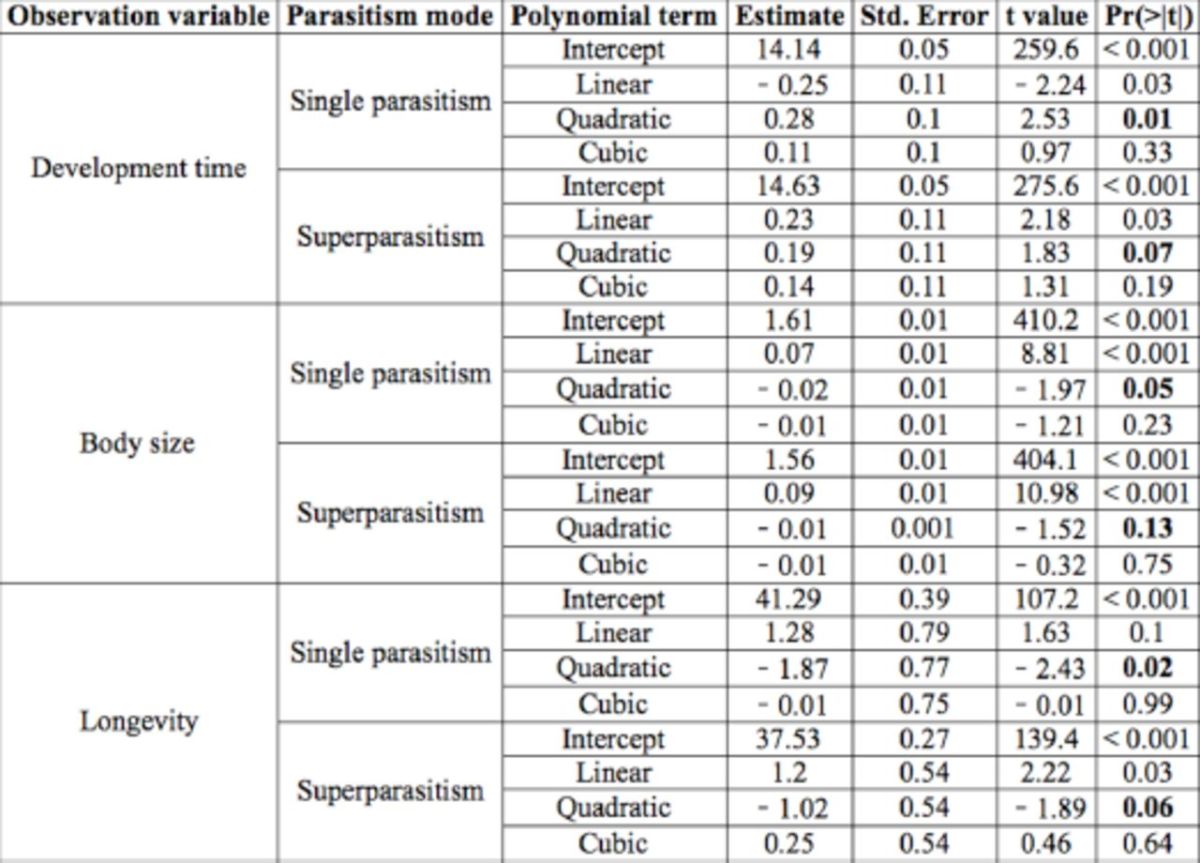
Results of orthogonal polynomial regression of life-history traits of *Meteorus pulchricornis* progeny parasitoids in relation to host instar singly parasitized (A) and self-superparasitized (B) as second–fifth instar larvae.

Bold values indicate significant or marginally significant differences.

Offspring adult parasitoids were significantly smaller in body size, as measured by hind tibia length, in self-superparasitized hosts than in singly parasitized hosts ( Figure 1C , D). Where the relationship between body size and host stage at oviposition was best fitted by a quadratic curvilinear function for offspring parasitoids engaged in single parasitism, it was best fitted only by a linear function for the counterparts involved in selfsuperparasitism ( Figure 1C , D; Table 1 ).

**Figure 2.**
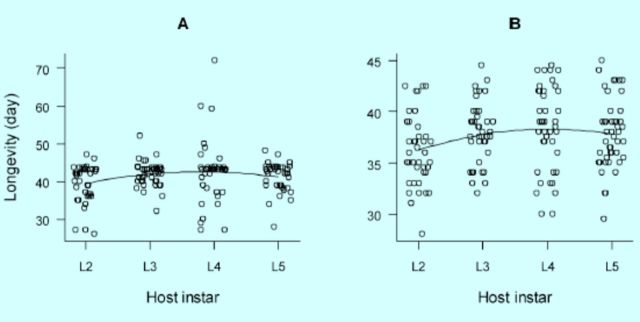
Longevity of *Meteorus pulchricornis* adults in relation to host instar singly parasitized (A) and self-superparasitized (B) as second–fifth instar larvae. High quality figures are available online.

Longevity of adults was shortened in self-superparasitized hosts as opposed to singly parasitized hosts ( Figure 2 ). Whereas there was a quadratic effect of host stage on longevity of parasitoids engaged in single parasitism, the quadratic effect was close to statistically significant for parasitoids involved in selfsuperparasitism ( *P* = 0.06) ( Figure 2A , B; Table 1 ).

**Figure 3:**
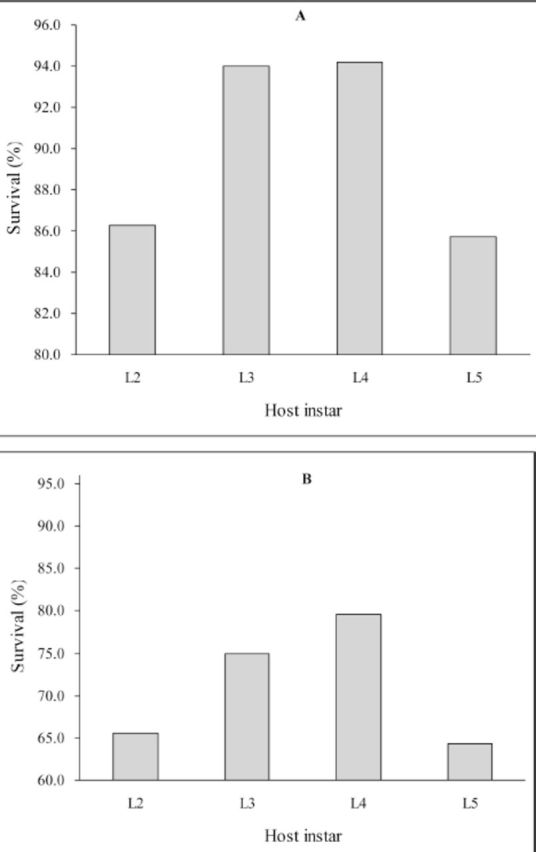
Percentage survival to adult emergence of *Meteorus pulchricornis* from hosts singly parasitized (A) and self-superparasitized (B) as second–fifth instar larvae. High quality figures are available online.

The survival of offspring parasitoids decreased by 15–21% in self-superparasitized hosts as compared to singly parasitized hosts ( Figure 3 ). The Cochran-Armitage test of trend supported the dome shaped trend in survival proportions across successive host stages at ovipositon for both single parasitism (χ ^2^ = 18.81, *P* <0.001) and selfsuperparasitism (χ2= 4.41, *P* = 0.04) ( Figure 3 ).

## Discussion

Our experiments show that selfsuperparasitism in *M. pulchricornis* had deleterious effects on the development of parasitoid progeny, leading to prolonged egg-to-adult emergence time, decreased survival to adult emergence, smaller body size, and shorter longevity of the emerging adults. The results provide data in support of some other studies, which ob served that superparasitism in solitary parasitoids delays the development of the progeny ( Eller et al. 1990 ; Harvey et al. 1993 ; Hegazi and Khafagi 2005 ; Chau and Maeto 2008 ; Tunca and Kilinger 2009 ), increases larval mortality ( Vinson and Spoka 1978 ; Tunca and Kilinger 2009 ), and results in offspring small in body size ( Harvey et al. 1993 ; Tunca and Kilinger 2009 ). The fitness costs in development of parasitoid progeny in a super parasitized host are generally assumed to be due to increased competition for limited resources among progeny inside the host (van Lenteren 1981; Waage and Godfray 1986). Moreover, some other competing behaviors may also bear fitness costs for the winning parasitoid. Chau and Maeto (2008) found, in the study of larval competition in *M. pulchricornis* , that fierce physical fighting between first-instar larvae oc18.81, *P* <0.001) and selfsuperparasitism (χ ^2^ curred inside the superparasitized host. However, some fitness costs for progeny parasitoids from superparasitism may be offset. For example, studies of the solitary aphid parasitoid *Monoctonus paulensis* show that progeny parasitoids are larger in body size in superparasitized aphid hosts than counterparts in singly parasitized aphids without a corresponding increase in development time ( Bai and Mackauer 1992 ; Mackauer and Chou 2001 ).

The results of this study show that fitness correlates in development of progeny parasitoids engaged in single parasitism do not linearly increase with host stage at oviposition. The analysis of orthogonal polynomial regression revealed a quadratic effect of host stage on development time, body size, and longevity of progeny parasitoids, respectively; the Cochran-Armitage test on survival proportions showed a dome-shaped trend from L2 to L3 hosts at oviposition. These results provide data in support of the findings not only with this parasitoid parasitizing other host species ( Liu and Li 2008 ), but also with other koinobiont parasitoids that exhibit developmental patterns illustrating clear trade-offs between body size, development time, and pre-adult mortality ( Smilowitz and Iwantsch 1975 ; Harvey et al. 1994 , 1999; Pandey and Singh 1999 ; Chau and Mackauer 2001 ; Elzinga et al. 2003 ; Harvey et al. 2004 ). Such domed fitness functions may be an adaptive response that optimizes several traits affecting parasitoid fitness ( Strand 2000 ).

The domed fitness functions, however, are demonstrated only in parasitoids engaged in single parasitism. There remains a need to know if these functions apply to the parasitoids engaged in superparasitism, in consideration of superparasitism as an adaptive strategy in parasitoids ( van Alphen and Visser 1990 ). The results of this study do not reveal a significantly quadratic function in development time, body size, and longevity of progeny parasitoids engaged in selfsuperparasitism with regard to host stages at oviposition, but support a quadratic trend in survival proportions across successive host stages. The discrepancy in developmental fitness functions between single parasitism and selfsuperparasitism, as exhibited in *M. pulchricornis,* may be explained by several factors. The eggs of parasitoids in several families (especially in the Braconidae) produce large cells, teratocytes, which are released to the host haemocoel when the parasitoid first instar ecloses ( Dahlman and Vinson 1993 ). Among other functions, teratocytes have the function of nutrition for developing parasitoid larvae. Bai and Mackauer (1992) reported that larvae of the solitary koinobiont endoparasitoid *Aphidius ervi* readily feed on them along with host tissues, and that the larger size of progeny emerging from superparasitized hosts compared with singly parasitized hosts may be a result of increased numbers of teratocytes circulating in the haemolymph of superparasitized hosts. Our study also provides some corroborative evidence for this factor. The body size of progeny initially increased with host instar, but rapidly declined when the host at oviposition was the final instar with single parasitism, whereas it linearly increased with host instar at oviposition with selfsuperparasitism.

Alternatively, the discrepancy may be explained by the same mechanism for the domed fitness functions in single parasitism. Strand (2000) suggested that with single parasitism, offspring fitness initially increases with host size, but as resources increase beyond the capacity of the parasitoid larva to consume them, fitness rapidly declines. We assumed that the resource threshold for the consumption of parasitoid larvae may be higher with selfsuperparasitism, so as to shift the increasing trend in some life-history traits, such as body size, with host size (or stage) at oviposition. In this respect, selfsuperparasitism may be an adaptive strategy in solitary koinobionts to exploit suboptimal host stages. This assumption has gained some support in the study of *Microplitis rufiventris* , where more parasitoid eggs survive to mature larvae when laid in the sixth-instar larva, the suboptimal stage for single parasitism, than the fifth-instar host, which is the optimal stage for single parasitism ( Khafagi and Hegazi 2005 ; Hegazi and Khafagi 2008 ). Furthermore, selfsuperparasitism may be enhanced by host sharing in solitary endoparasitoids toward the evolution of gregariousness over time (Rid-dich 2002; Khafagi and Hegazi 2005 ). Pexton and Mayhew (2005) reported that competition for hosts may have contributed to the evolution of gregarious development in the braconid genus *Aphaereta* .
